# Nordic research on health inequalities: A scoping review of empirical studies published in *Scandinavian Journal of Public Health* 2000–2021

**DOI:** 10.1177/14034948221101304

**Published:** 2022-06-22

**Authors:** Jon Ivar Elstad, Kristian Heggebø, Espen Dahl

**Affiliations:** 1 NOVA, Centre for Welfare and Labour Research, Oslo Metropolitan University, Norway; 2 Department of Social Work, Child Welfare and Social Policy, Oslo Metropolitan University, Norway

**Keywords:** Systematic review, health inequities, health inequality theory, research methods

## Abstract

**Aims:**

An important task for the *Scandinavian Journal of Public Health* is to address health inequality topics. This scoping review characterises Nordic empirical studies within this research field, published 2000–2021 by the *Scandinavian Journal of Public Health*.

**Methods:**

Original empirical research studies using data from Denmark, Finland, Iceland, Norway and/or Sweden, which linked differences in health or health-related aspects to socioeconomic positions, immigrant status, family structures and/or residential areas, were included in the review. The initial search in the Web of Science article database resulted in 294 possibly relevant articles, and 171 were judged to comply with our criteria.

**Results:**

Only one study was based on qualitative data, while all others used either surveys or register data, or both in combination. A wide variety of outcomes was addressed. Most studies had a social causation design, but 16 studies analysed health-related mobility processes and four reported intervention results. The most common statistical method was logistic regression. Poisson, Cox and ordinary least squares regression were less used. Few studies engaged explicitly with health inequality theories or with rigorous causality designs.

**Conclusions:**

**The empirical health inequality studies published by the *Scandinavian Journal of Public Health* are rich sources for knowledge on a large array of health and health-related inequalities in Nordic countries. Drawbacks are underuse of qualitative data, few theoretical discussions and lack of studies assessing effects of interventions and policies.**

## Introduction

Health inequalities – the ubiquitous pattern that morbidity and mortality vary systematically with placement in social hierarchies – have always been central for public health [1], but such topics did not draw much attention in Nordic countries before the 1980s [2,3]. Decades with rising material standards, expanding public welfare and egalitarian beliefs had nurtured ideas that social class differences in health would gradually become a phenomenon of the past.

In the last quarter of the 20^th^ century, however, various studies showed that social inequalities in disease and death also persisted in the Nordic welfare states [4,5]. A cross-country comparative study, indicating that educational differences in health were not smaller in these countries than elsewhere in Western Europe, was an eye-opener [6]. The rising awareness of enduring, socially structured, inequalities in mortality, disease and illness made health inequalities a prominent policy issue in England, the Netherlands and the Nordic countries during the 1990s and onwards [7,8]. Research funding became available, researchers were attracted by new opportunities, and numerous empirical studies came to light.

On this background, a given task for the *Scandinavian Journal of Public Health* (SJPH) has been to publish health inequality research from the Nordic countries. The ‘Aims and scope’ statement, although broad (see https://journals.sagepub.com/aims-scope/SJP), emphasises the journal’s commitment to promote public health research in these countries. This article presents a scoping review of Nordic empirical studies on health inequalities published in SJPH since the turn of the millennium. Unlike systematic reviews which attempt to collect, assess and synthesise available evidence into well-substantiated conclusions about causes or the effectiveness of treatments, a scoping review will typically restrict itself to map the scientific literature in a particular area [9,10]. Its goal will be to describe issues, theories, concepts and research methods, as well as pointing to relevant topics not addressed by the existing literature. A scoping review is ‘an ideal tool to determine the scope or coverage of a body of literature on a given topic [and] the volume of literature and studies available’ [11].

Accordingly, the purpose of the present article is not to evaluate and summarise SJPH article findings on health inequalities in Nordic countries. As will be seen below, this is hardly feasible in a single article due to the multitude of studies and great diversity in topics and approaches. Rather, the purpose is to survey, both quantitatively and qualitatively, how Nordic health inequality research has surfaced in the SJPH. We characterise the research questions, methods and theories that appear in this literature, and draw attention to changes over time, to knowledge gaps and topics and to approaches that seem unduly absent. This will hopefully be interesting for health inequality researchers, serve as useful input for discussions about SJPH’s future publishing, provide an overview of available contributions which can be used for more targeted summaries, and function as a reference for examinations of health inequality research in other journals. When designing this study, we have followed broadly the recommended guidelines and checklists for scoping reviews [10,11].

## Methods

### Selection of articles

The reviewed articles were located by means of the article database Web of Science Core Collection (Clarivate). This database lists titles, authors and (if available) abstracts for all items published by SJPH since 2000. It has also information on articles published in the predecessor *Scandinavian Journal of Social Medicine* (SJPH changed its name in 1999). As preliminary analyses found few relevant articles in the predecessor journal, and Nordic health inequality research expanded considerably in the new millennium, this review concentrates on studies published during 2000–2021.

Our goal has been to review all original empirical health inequality studies published in this period, based on data collected in Nordic countries, i.e. Denmark, Finland, Iceland, Norway and/or Sweden. Thus, we excluded commentaries, reviews of previous empirical studies, and articles only addressing policy, organisational, theoretical or methodological questions without original empirical analyses. Also, the small number of empirical studies comparing Nordic countries with non-Nordic countries were excluded.

A further criterion was that studies should either employ data about mortality, disease or illness, or about clearly health-related topics such as healthcare utilisation and important precursor of illness or disease (e.g. smoking, adverse working conditions, economic poverty). How to define ‘clearly health-related’ is obviously debateable. To exemplify our judgements, sense of coherence, coping and feelings of severe time pressure were included, while health literacy, private health insurance and parent-infant relations were excluded.

Furthermore, studies should clearly relate differences in health or health-related topics to differences between social positions. Social positions were generally defined as different placements in a social structure that can be hierarchically ordered according to differences in resources, status or power. Typical examples are the educational hierarchy, income level, the occupational structure and the labour market (e.g. employed, unemployed, homeworker, disability pension, etc.). Social positions were also indicated by terms such as social class, socioeconomic status (SES), socioeconomic group (SEG) or Family Affluence Scale [12]. Also immigrant status, majority/minority belonging, residential areas (if ranked by, for example, average income or unemployment level), and family situation (for example married, divorced, single parent, etc.) were considered as social positions in a hierarchical social structure. Studies on gender differences were included when linked to employment, working conditions or income. Studies which only examined health differences according to gender, housing tenure or age were not included. Moreover, if social positions had a very marginal role in the analyses, for instance when neglected in study conclusions or only used for statistical controls without reporting coefficients, the article was excluded from the review.

Using these criteria, we sought to select all relevant SJPH articles published (or made available as early access) during 2000–2021. First, we located possibly relevant articles in the Web of Science Core Collection (Clarivate) database by means of the search terms ‘(health or mortality or death or disease* or illness* or morbidity) and (equal* or inequ* or disparit* or unequ* or equit*) and (Denmark or Danish or Finland or Finnish or Iceland* or Norway or Norwegian or Sweden or Swedish)’, applied to titles, abstracts and keywords. This resulted in 294 articles. Further selection was made by reading abstracts and, in case of doubt, consulting the full article. This was mainly done by the first author, but all three authors examined 30 articles in order to clarify criteria and reach decisions in case of ambiguities. We decided that 127 of the 294 articles should not be included because they were review articles, solely addressed policy issues without empirical data or fell outside for other reasons. Accordingly, 167 articles remained but from reference lists we discovered four more relevant articles which had not been spotted by our search terms. Thus, our final sample includes 171 articles.

### Analyses

The selected articles were classified according to publishing year, country setting, data sources, main outcomes and types of social positions used in the analyses. This classification was based on abstracts and by consulting full articles when in doubt. Results are reported for two 11-year periods (2000–2010 and 2011–2021) in order to indicate changes over time.

Furthermore, we examined methods and statistical tools by reading the methods sections of all 171 articles and inspecting their tables and figures. Lastly, how health equality theories were used was assessed in a similar way by reading the introduction and discussion sections of all the included articles.

The 171 articles selected for this review are listed in the online Supplemental Material Appendix. When referring to reviewed articles, we use the article number as indicated in the Supplemental Material Appendix.

## Results

### Quantitative analyses

Among the 171 articles, 61 were published during 2000–2010 and 110 during 2011–2021. The increase reflects the growth in Nordic health inequality research, but also overall growth in SJPH publishing (1097 articles and other items, all types included, were published during 2000–2010, rising to 1690 during 2011–2021). More than a third of the studies (36.3%) originated from Sweden, 21.1% from Denmark, 15.8% from both Finland and Norway, only five studies were from Iceland, while 14 studies used data from two or more Nordic countries.

Table I shows that data were mostly collected not only by surveys (e.g. face-to-face interviewing, telephone interviews, postal questionnaires) but also frequently from registers (administrative records on deaths, death causes, disability pension, patient registers of hospitalisation, cancers, diabetes). Only one study was based solely on qualitative interviews (online Supplemental Material Appendix 131). The proportion of studies based on single cross-sectional surveys declined from the first to the second period, while the proportion of panel surveys and surveys combined with registers rose. The latter type typically linked a baseline survey (sometimes including data from laboratory tests) to follow-up register data on, for instance, mortality, healthcare use or disability pensioning.

**Table I.** Classification of main data sources of 171 *Scandinavian Journal of Public Health* (SJPH) articles.

Data sources/period	2000–2010		2011–2021		Total	
	*n*	%	*n*	%	*n*	%
Cross-sectional surveys	18	29.5	24	21.8	42	24.6
Repeated cross-sectional surveys	8	13.1	9	8.2	17	9.9
Panel surveys	6	9.8	17	15.5	23	13.5
Administrative register, census	19	31.1	34	30.9	53	31.0
Survey plus register	10	16.4	25	22.7	35	20.5
Qualitative interviews	0	0	1	1.0	1	0.6
Total	61	100.0	110	100.0	171	100.0

A sizeable proportion of the articles were based on data collections organised by large, longitudinal projects. Both data from the Norwegian Trøndelag Health Study (HUNT) and Swedish Surveys of Living Conditions data were used in nine articles. Nordic branches of the long-lasting multi-country project Health Behaviour in School-Age Children (HBSC) delivered data for eight articles. Other examples of such data collection projects were the Helsinki Municipal Employee Study, Scania Public Health Cohort, Northern Swedish Cohort, and Swedish Health on Equal Terms project.

The outcomes which the studies sought to illuminate were very diverse (Table II), but some topics occurred relatively often. We located 33 articles addressing different forms of mortality, 20 articles on self-rated health and 11 studies on smoking or on trajectories into disability pension. Small clusters of studies analysed birth weight, obesity and healthy life expectancy. However, many studies examined a unique outcome not addressed by any other, and the categories in Table II often have a very varied content. The 30 articles on healthcare utilisation, for instance, investigated emergency rooms, overall hospitalisation, hospitalisation for specific diseases, elective surgery, hypertension care, fertility treatment, dental care, vaccinations and various other healthcare types. Psychological conditions covered severe depression, unspecified self-reported psychological malaise, coping and sense of coherence.

**Table II.** Main outcomes of 171 *Scandinavian Journal of Public Health* (SJPH) articles.

Outcomes/period	2000–2010		2011–2021		Total	
	*n*	%	*n*	%	*n*	%
Mortality, all-cause and cause-specific deaths	16	21.9	17	13.1	33	16.3
Self-rated health	10	13.7	10	7.7	20	9.9
Longstanding/chronic conditions	10	13.7	13	10.0	23	11.3
Psychological conditions	1	1.4	16	12.3	17	8.4
Body mass, obesity	2	2.7	4	3.1	6	3.0
Healthy life expectancy	2	2.7	5	3.8	7	3.4
Low birth weight, infant mortality.	2	2.7	1	0.8	3	1.5
Disability pensioning, retirement	4	5.5	7	5.4	11	5.4
Sickness absence	2	2.7	1	0.8	3	1.5
Smoking or other health behaviours	9	12.3	16	12.3	25	12.3
Health effects on careers, financial stress etc.	4	5.5	6	4.6	10	4.9
Healthcare utilisation	7	9.6	23	17.7	30	14.8
Other topics	4	5.5	5	3.8	9	4.4
Intervention, policy effects on health inequalities	0	0.0	6	4.5	6	3.0
Total^a^ =100.0%	73	100.0	130	100.0	203	100.0

a Quite a few studies addressed several outcomes; number of outcomes (=203) exceeds number of studies (=171).

Table II indicates a shift over time towards more interest in psychological outcomes, and a movement from studies of generic outcomes (e.g. self-rated health, all-cause mortality, overall health service use) to more specific outcomes such as coping status in a particular patient category (Supplemental Material Appendix 106) or fracture-related mortality (Supplemental Material Appendix 87). The dominant approach has been to investigate how social positions precede health or health determinants. Nonetheless, we found 10 studies addressing how health influenced social positions in terms of, for example, educational attainment or financial situation (Supplemental Material Appendix 15, 170). Also, several studies analysed how disease was followed by labour market exit and disability pension.

We found four studies which explicitly analysed interventions aiming at reducing health inequalities (Supplemental Material Appendix 9, 14, 135, 141), while two studies (Supplemental Material Appendix 10, 41) assessed health inequality effects of macro-policy variations. None of these studies were published in the first period; five of them were published as recently as 2018–2021.

Often two, three, or even more types of social positions were used in the same study (Table III). Their placement in the analysis varied a lot. Some studies, as noted above, analysed health effects on attainment of social positions, but the majority designated social positions as a direct, proximate or distal factor for a health or health-related outcome. Social positions were also used as modifiers in a few articles, for example a study on how higher income could ‘buffer’ against unhealthy effects of high body mass index (BMI) (Supplemental Material Appendix 135).

**Table III.** Social position classifications used in 171 *Scandinavian Journal of Public Health* (SJPH) articles.

Types of social positions/period	2000–2010		2011–2021		Total	
	*n*	%	*n*	%	*n*	%
Education	24	25.5	48	32.4	72	29.8
Occupational class	24	25.5	18	12.2	42	17.4
Income	14	14.9	23	15.5	37	15.3
Labour market position	15	16.0	12	8.1	27	11.2
Other socioeconomic classifications^a^	5	5.3	9	6.1	14	5.8
Subjective assessments^b^	2	2.1	14	9.5	16	6.6
Marital status, family structure	4	4.3	9	6.1	13	5.4
Immigrant status, minorities	5	5.3	5	3.4	10	4.1
Geographical divisions	1	1.1	10	6.8	11	4.5
Total *n* (types social positions)^c^	94	100.0	148	100.0	242	100.0

a Erikson, Goldthorpe, Portocarero (EGP) social class, Swedish socioeconomic classification, socioeconomic status (SES), socioeconomic group (SEG), typically constructed from several indicators.

b E.g. self-reported financial or work-related stress, Family Affluence Scale.

c Many studies used two or more types of social position in the analyses.

The most used social positions were education and occupation. In the first period, these two were used equally often, but education predominated in the latter period. Income, measured in different ways (self-reported, taxation registers, household-equalised disposable income, etc.) was also often used but wealth indicators were practically absent. The labour market position category covers both the employed/not-employed distinction and various forms of non-employment such as unemployed, homeworker, retired or disability pension. Some studies assessed gender differences, for instance with respect to health effects of unemployment (Supplemental Material Appendix 57) or trends in educational health inequalities (Supplemental Material Appendix 118). Studies on ethnic minorities, immigrant categories or geographical areas were relatively few.

### Methodological approach – statistical methods

Since there was only one qualitative study, an assessment of methodological approaches will in practice focus on statistical methods. A large variety of techniques was used. Pearson’s correlation coefficients, Kaplan-Meier curves, ordinary least squares (OLS) regression and multilevel models are but a few examples. The most common method by far was logistic regression, which appeared in almost 50% of the articles. Cox proportional hazard models were used in 29 studies, while Poisson regression models appeared in about one-tenth of the articles. Various types of indexes, typically slope index of inequality (SII), relative index of inequality (RII) or concentration index, appeared in roughly one-tenth of the studies. Weighted least squares appeared two times (Supplemental Material Appendix 140, 160) whereas one study used multiple correspondence analysis (Supplemental Material Appendix 121).

An observation is that statistical techniques suitable for establishing causal inference, such as individual-level fixed effects or propensity score matching, were practically absent. Exceptions include one difference-in-difference analysis of smoking among adolescents (Supplemental Material Appendix 14) and two studies of educational effects on cardiovascular disease which controlled for shared family factors by a sibling design (Supplemental Material Appendix 76, 145). As health inequalities often arise from cumulative exposures over the entire life course, employing a (counterfactual) causal inference framework is challenging, for instance because randomised controlled trials can hardly be utilised. Nevertheless, it was surprising that only very few studies had attempted to pursue causal inference issues.

Some change over time in the use of statistical techniques occurred. During the first decade, age-standardised prevalence and prevalence rates were common. Indexes gained more popularity in 2011–2021. Logistic regression and Cox proportional hazard models were frequently used throughout the entire analysed period. There seemed to be a slight change over time in how statistical models were set up. More parsimonious models (e.g. only adjusting for age, gender and marital status) were common in the first period. Later, the number of included variables has grown and the models have become more complex.

In general, the studies seem to be methodologically sound. Study samples were most often described thoroughly. Empirical results from different model specifications were shown, often accompanied by nuanced interpretations of emerging differences and similarities. Most studies also tended to include sober discussions of methodological strengths and weaknesses, and many articles pointed to future directions for studying empirically the addressed topic.

Nonetheless, there are debatable aspects regarding how the statistical methods were presented and used. In a handful of the articles, it was difficult to figure out exactly what was the type of regression that had been estimated. Without a clear description of what statistical techniques were used, and which variables and covariates were included in model specifications, findings are difficult to evaluate and reproducibility is hindered.

We also noted studies which seemed to neglect potential difficulties if many covariates were included in the same statistical model. This may entail over-adjustment which complicates interpretation of the explanatory variable coefficients. Moreover, including several variables that correlate to a high extent might lead to multicollinearity which further aggravates interpretation difficulties. If, for instance, education, occupation and income are simultaneously included in a model, the meaning of the coefficients is uncertain. Such difficulties can, at least to some extent, be circumvented if crude associations between explanatory variables and outcomes are reported as well. Fortunately, many studies took care to reduce such difficulties by reporting univariate distributions, crude bivariate associations, and sometimes associations minimally adjusted, for example, by age.

A related issue is when results are compared across dissimilar model specifications, for example in gender split analyses. Some studies only included covariates in the multivariate model if the covariate was significantly associated with the outcome measure(s) in the bivariate analysis, resulting in different model specifications for the subgroups in question (and interpretation problems). Many studies used logistic regression and contrasted odds ratios (ORs) between different samples (e.g. two different survey years) or across differing model specifications (e.g. by stepwise inclusion of covariates). By now, the risk of misinterpretation when comparing coefficients derived from different logit and probit models should be well-known [13,14]. Only three of the reviewed studies that used logistic regression computed average marginal effects (AMEs), used the Karlson-Holm-Breen (KHB) method or took any other steps towards making comparisons more robust. In a similar vein, data clustering, for example where people are followed longitudinally or where pupils are clustered in schools and municipalities, can lead to biased standard errors. This topic was seldom addressed in the reviewed studies, although robust standard errors were sometimes reported (Supplemental Material Appendix 82, 118).

An additional observation is that in a few cases, very similar articles with practically identical methods were published in SJPH by (some of) the same authors, only a few years apart. Highly overlapping articles are not necessarily problematic, and it may be rewarding to run the same analyses again on newer data sources. Also, replication of previous studies should be encouraged. The overlap is more problematic, however, if a new updated study does not explicitly acknowledge the original study but is framed as an original contribution.

### Theoretical references

In order to assess how ‘theory’ has been used in the 171 articles, we may note that ‘theory’ is often understood in two quite different ways [15]. One conception is a systematic theoretical model that allows one to derive hypotheses that can be tested against empirical evidence. The primary aim is to provide a substantive theory, for instance about the ways in which social class and health are related. Another notion of ‘theory’ refers to a conceptual framework for empirical research which establishes analytical tools for the systematic generation of scientific knowledge. Here, ‘theory’ is a way of looking at social and health-related phenomena, which may be described by terms such as 'paradigm', 'heuristic device' or 'conceptual framework'. Other concepts, for instance variants which highlight how theory can guide actions towards social change, are also found in discussions on health inequality theories [16].

Over the past decades, a number of theories and theoretical models have been developed to explain health inequalities. Most current theories can be subsumed under the perspective of social determinants of health (SDoH). SDoH approaches can be categorised into four main groups: material, behavioural, psychosocial and the life course approach [17]. The latter examines both how environments will affect health across a person’s lifetime, as well as the role of health-related social mobility, for instance in terms of how ill health may hinder educational attainment or push individuals out of paid employment.

On reading the 171 articles, a first observation is that a number of them do not refer to theory at all. As in the tradition of medical research, the articles focus on new research findings, with no or little emphasis on theory as a motivation for doing the research or for interpreting the results. However, even if many articles lack theoretical reflections, theoretical reasoning is implied. This can be derived from the choice of research questions, selection of variables and the way in which the data analysis is set up. Health-related behaviours like smoking or physical activity are fairly often used as outcome variables, implying that differences or changes in health-related behaviours have consequences for health outcomes and their social distribution. In other articles, health behaviour is assigned as a mediating variable without much justification – often, it seems it is taken for given that health behaviour operates as a mediating variable between socioeconomic positions and health.

A second observation in line with the preceding one is that only one of the 171 articles used the word ‘theory’ in the abstract (the only qualitative study, Supplemental Material Appendix 131, referred to ‘grounded theory methods’). When reading the main texts of the articles, it appeared that when ‘theory’ is referred to, it is usually as a heuristic device as defined above, and not in order to do more rigorous hypothesis testing. Often, a reference to one or more of the four above-mentioned SDoH perspectives was made in the introduction section, for instance psychosocial stress or health-related behaviours. Theory was also used when findings were interpreted ad-hoc with reference to general theories or to mechanisms such as stress, hazardous exposures, health behaviours or known social or biological risk factors.

All the four different approaches within the SDoH perspective alluded to above were represented in the articles. However, health behaviours were most often examined, often with no reference to other major explanations for health inequalities. For this reason, knowledge of relationships between health behaviours and health inequalities has become well developed through the SJPH studies, while less knowledge has emerged about how the health behaviour model relates to other models in the SDoH perspective.

Nonetheless, references to life course or psychosocial models were also made in several articles, but few studies addressed the materialist model directly. Sometimes, studies of income effects on health (e.g. Supplemental Material Appendix 46, 53, 86, 107, 118, 154) suggested material pathways, i.e. low income leading to poor housing, insufficient nutrition and other unhealthy material disadvantages. Usually, however, direct measurements of material environments were lacking, and income effects were seldom interpreted by means of the psychosocial model. We found only one study using measurements of physical working conditions (Supplemental Material Appendix 52), but low status occupation was sometimes used as a proxy measurement of hazardous and physically demanding work (e.g. Supplemental Material Appendix 55).

This leads to the third observation that proximate, rather than distal, factors were the focus in many of the articles. Research on ‘the causes of the causes’, such as policies, policy regimes or structural forces and changes, seldom occurred. It is notable that proximate causes like smoking were rarely, if at all, interpreted in light of macro phenomena like tobacco control policies or secular trends in health-related behaviours. Yet, as mentioned above, two studies (Supplemental Material Appendix 10, 41) linked healthcare organising and welfare policies on a macro level to health inequalities, and a few others (e.g. Supplemental Material Appendix 33, 97) interpreted results in light of structural and contextual conditions.

It is often called for knowledge on ‘what works’ to reduce health inequalities, i.e. a demand for evaluations of interventions undertaken to achieve this aim. The four intervention studies (Supplemental Material Appendix 9, 14, 135, 141) were all directed towards change in health-related behaviours, e.g. consumption of sweets, physical activity and smoking habits. This implies that knowledge on what works is limited to just one of the SDoH explanatory models. Hence, in this field theories of change are much needed, and so are interventions and trials that do not only address health behaviour and health-related habits.

Underlying virtually all the included studies of utilisation of healthcare was an equity perspective, i.e. that use of healthcare should above all be determined by ‘need’. Implicitly or explicitly, Andersen’s behavioural model of health services utilization [18] was applied, i.e. use is determined by predisposing factors, enabling factors and need. Accordingly, studies of healthcare use (e.g. Supplemental Material Appendix 3, 37) tried to control for need and examined whether socioeconomic positions had additional effects. Lastly, we may add that references to national contexts, often made in the articles, imply theory-related presuppositions. Authors regularly mentioned the Nordic, ‘social-democratic’ welfare state, its egalitarian ethos, compressed income distribution, generous social benefits and universal coverage of health services, as a background for choice of topics and discussion of findings.

## Discussion

The 171 reviewed articles constitute without doubt a very rich source of information about health inequalities in Nordic countries. Many topics have been addressed and many types of data and statistical approaches have been utilised. Findings provide useful and interesting knowledge and are fruitful points of departure for future research. We also note that SJPH publishing of Nordic empirical health inequality studies has increased from 5.5 per year during 2000–2010 to 10.0 per year during 2011–2021. Psychology-related outcomes were more in focus in the latter period than in the first; the proportion of studies which classified samples according to occupation has declined; and the statistical models utilised by researchers have become more complex.

Our aim is not to summarise findings, but we will nevertheless point to two conspicuous patterns. First, trends studies tended to report either stable or widening health inequalities over time (Supplemental Material Appendix 23, 68, 151, 164, 167) but few instances of narrowing inequalities (although see Supplemental Material Appendix 153). Second, almost all studies on how healthcare services were distributed, discovered social, income or educational inequalities (e.g. Supplemental Material Appendix 3, 52, 164). Thus, tax-funded healthcare, practically universal coverage and small co-payments appear as insufficient for hindering inequities in health service provision.

Our review uncovered some ‘blind spots’ in the health inequality research published by SJPH. Although some studies examined health-related social mobility processes, in particular exits from employment, this phenomenon may deserve more attention. Likewise, only scattered examples of studies on how macro-factors and social policy reforms impact on health inequalities could be found. Moreover, we had objections to how statistical techniques were used in some studies, but a more salient criticism is the glaring lack of qualitative studies. Various topics, such as why social inequalities in health behaviours arise, why privileged individuals receive better healthcare, etc. may be better understood if also addressed by qualitative studies. Nordic researchers have carried out such studies, but SJPH is apparently not the preferred outlet for such research.

A further observation is that SJPH studies seldom examined possible causal processes in a stringent manner. Causal inference is indisputably complex, but that should not prevent authors from attempting to meet the challenge. Substantiated causal claims are needed when trying to clarify the distinction between health inequalities which ‘only’ are differences, and those which are ‘true’ inequities, i.e. unfair and avoidable health differences. Related to the causality issue is the observation that the four intervention studies had a limited focus, as they only addressed potential change in health-related behaviour. As to how health inequalities can be reduced, there are huge knowledge gaps both regarding theory and methods. In the empirical studies reviewed here, theoretical perspectives are rarely elaborated. As an illustration, the ‘fundamental cause theory’ [19] has inspired health inequality debates in recent decades, but only one of the 171 articles had a brief reference to this theory (Supplemental Material Appendix 112). However, to be fair, SJPH has also published articles which address conceptual and theoretical issues, for instance articles not included in our review since they had no empirical research [20–23].

### Strengths and limitations

The present article has probably succeeded in surveying the entire body of Nordic empirical health inequality studies published in SJPH during 2000–2021.We have adhered to guidelines for scoping reviews, implying that the ambition is not to summarise and evaluate critically the substantive findings, but to characterise the SJPH studies with respect to volume, topics, data sources, methods, theoretical references and developments over time. Another ambition has been to identify knowledge gaps and unaddressed topics. The analyses, made by combining quantitative analyses with qualitative approaches, have hopefully provided insights which may be interesting both for the research community and for SJPH’s future editorial policy.

As to limitations: in order to be included in the review studies were required to analyse ‘clearly health-related’ topics and link them distinctly to social positions. Due to the diversity of topics and approaches, some studies were difficult to categorise. We made decisions after collectively discussing ambiguous cases but cannot preclude that other researchers would sometimes conclude otherwise. Moreover, we cannot be certain that our initial search terms, which resulted in a large output (294 articles), spotted all SJPH studies which would possibly be relevant for this scoping review.

A more general limitation is that the present article only addresses studies published in SJPH. Whether our results apply to Nordic health inequality studies in general cannot be determined by our study. An interesting topic would be to compare SJPH’s publishing profile with international health inequality research in general, as well as publishing profiles of other public health journals, but this could not be done by the present study.

## Conclusion

Overall, SJPH has, during the two first decades of this century, published a large number of solid, well-done, empirical analyses on a number of health inequality topics in the Nordic countries. The published literature provides rich sources for insight into the Nordic situation. This scoping review of 171 articles points nevertheless to some ‘blind spots’, such as an absence of qualitative studies, little attention to health inequality theories and a lack of studies of effects of interventions and policy changes on health inequalities.

## Supplemental Material

sj-docx-1-sjp-10.1177_14034948221101304 – Supplemental material for Nordic research on health inequalities: A scoping review of empirical studies published in Scandinavian Journal of Public Health 2000–2021 Click here for additional data file. Supplemental material, sj-docx-1-sjp-10.1177_14034948221101304 for Nordic research on health inequalities: A scoping review of empirical studies published in Scandinavian Journal of Public Health 2000–2021 by Jon Ivar Elstad, Kristian Heggebø and Espen Dahl in Scandinavian Journal of Public Health

## References

[1] Berkman LF Kawachi I. A historical framework for social epidemiology. In: Berkman LF Kawachi I (eds) Social epidemiology. Oxford: Oxford University Press, 2000, pp.3-12.

[2] Dahl E. Sosial ulikhet i helse: Artefakter eller seleksjon? [Social inequality in health? Artefacts or selection?]. PhD Thesis, University of Oslo, Department of sociology. Oslo: FAFO-rapport 170, 1994.

[3] Lahelma E Lundberg O Manderbacka K , et al. Changing health inequalities in the Nordic countries? Scand J Public Health 2001; 29 (Suppl.55): 1-5. 11482792

[4] Lundberg O. Den ojämlika ohälsan. Om klass-och könskillnader i sjuklighet [Inequality in ill health. On class and sex differences in illness]. Stockholm: Swedish Institute for Social Research, 1990.

[5] Lahelma E Manderbacka K Rahkonen O , et al. Ill-health and its social patterning in Finland, Norway and Sweden. Research Reports 27. Helsinki: National Research and Development Centre for Welfare and Health (STAKES), 1993.

[6] Mackenbach JP Kunst AE Cavelaars AEJM , et al. Socioeconomic inequalities in morbidity and mortality in western Europe. Lancet 1997; 349: 1655-1659. 9186383 10.1016/s0140-6736(96)07226-1

[7] Mackenbach JP. Health inequalities. Persistence and change in European welfare states. Oxford: Oxford University Press, 2019.

[8] Dahl E Lie M. Policies to tackle health inequalities in Norway: From laggard to pioneer? Int J Health Serv 2009; 39: 509-523. 19771953 10.2190/HS.39.3.e

[9] PRISMA. PRISMA for scoping reviews, http://www.prisma-statement.org/Extensions/ScopingReviews (2021, accessed 18 March 2022).

[10] Rethlefsen ML Kirtley S Waffenschmidt S , et al. PRISMA-S: An extension to the PRISMA statement for reporting literature searches in systematic reviews. Systematic Reviews 2021; 10:39. 10.5195/jmla.2021.962 PMC8270366 34285662

[11] Munn Z Peters MDJ Stern C , et al. Systematic review or scoping review? Guidance for authors when choosing between a systematic or scoping review approach. BMC Med Res Methodol 2018; 18:143. 10.1186/s12874-018-0611-x PMC6245623 30453902

[12] Torsheim T Nygren JM Rasmussen M , et al. Social inequalities in self-rated health: A comparative cross-national study among 32,560 Nordic adolescents. Scand J Public Health 2018; 46: 150-156. 29039236 10.1177/1403494817734733

[13] Allison PD. Comparing logit and probit coefficients across groups. Sociol Method Res 1999; 28: 186-208.

[14] Mood C. Logistic regression: Why we cannot do what we think we can do, and what we can do about it. Eur Sociol Rev 2010; 26: 67-82.

[15] Mouzelis N. Sociological theory: What went wrong? Diagnosis and remedies. London: Routledge, 1995.

[16] Smith KE Schrecker T. Theorising health inequalities: Introduction to a double special issue. Soc Theory Health 2015; 13: 219-226.

[17] Bartley M. Health inequality: An introduction to theories, concepts and methods. Cambridge, UK: Polity Press, 2017.

[18] Aday LU Andersen R. A framework for the study of access to medical care. Health Serv Res 1974; 9: 208-220. 4436074 PMC1071804

[19] Link BG Phelan J. Social conditions as fundamental causes of disease. J Health Soc Behav 1995; 35: 80-94. 7560851

[20] Vallgarda S. Ethics, equality and evidence in health promotion Danish guidelines for municipalities. Scand J Public Health 2014; 42: 337-343. 24608091 10.1177/1403494814525007

[21] Oversveen E Rydland HT Bambra C , et al. Rethinking the relationship between socio-economic status and health: Making the case for sociological theory in health inequality research. Scand J Public Health 2017; 45: 103-112. 28078944 10.1177/1403494816686711

[22] Weiss D Eikemo TA. Technological innovations and the rise of social inequalities in health. Scand J Public Health 2017; 45: 714-719. 29162014 10.1177/1403494817711371

[23] Veenstra G Abel T. Capital interplays and social inequalities in health. Scand J Public Health 2019; 47: 631-634. 30672396 10.1177/1403494818824436

