# Supplementary material for: Nordic research on health inequalities: A scoping review of empirical studies published in Scandinavian Journal of Public Health 2000–2021
Source: Scand J Public Health. 2022 Jun 22;50(7):843–51. doi: 10.1177/14034948221101304 (PMC9578093; doi:10.1177/14034948221101304)
Supplement: sj-docx-1-sjp-10.1177_14034948221101304 – Supplemental material for Nordic research on health inequalities: A scoping review of empirical studies published in Scandinavian Journal of Public Health 2000–2021 [file sj-docx-1-sjp-10.1177_14034948221101304.docx]

**Online Appendix:**

**171 studies analysed by “Nordic research on health inequalities: A scoping review of empirical studies published in SJPH 2000—2021”**

| **NR** | **AUTHOR(S)** | **YEAR** |
| --- | --- | --- |
| 1 | Abebe, D. S., et al. (2016). "Municipal-level differences in depressive symptoms among adolescents in Norway: Results from the cross-national Ungdata study." Scandinavian Journal of Public Health **44**(1): 47-54. | 2016 |
| 2 | Af Sillen, U., et al. (2005). "Self-rated health in relation to age and gender: Influence on mortality risk in the Malmo Preventive Project." Scandinavian Journal of Public Health **33**(3): 183-189. | 2005 |
| 3 | Agerholm, J., et al. (2013). "Socioeconomic differences in healthcare utilization, with and without adjustment for need: An example from Stockholm, Sweden." Scandinavian Journal of Public Health **41**(3): 318-325. | 2013 |
| 4 | Airaksinen, J., et al. (2016). "Moving on: How depressive symptoms, social support, and health behaviors predict residential mobility." Scandinavian Journal of Public Health **44**(4): 394-401. | 2016 |
| 5 | Al-Emrani, F., et al. (2013). "The influences of childhood and adult socioeconomic position on body mass index: A longitudinal Swedish cohort study." Scandinavian Journal of Public Health **41**(5): 463-469. | 2013 |
| 6 | Andersen, I., et al. (2008). "Does job strain mediate the effect of socioeconomic group on smoking behaviour? The impact of different health policies in Denmark and Sweden." Scandinavian Journal of Public Health **36**(6): 598-606. | 2008 |
| 7 | Andersen, I., et al. (2011). "Increasing prevalence of depression from 2000 to 2006." Scandinavian Journal of Public Health **39**(8): 857-863. | 2011 |
| 8 | Andersen, M. B., et al. (2020). "Social inequality in lifestyle, motivation to change lifestyle and received health advice in individuals with diabetes: A nationwide study." Scandinavian Journal of Public Health **48**(8): 847-854. | 2020 |
| 9 | Anttila, J., et al. (2019). "School-level changes in factors related to oral health inequalities after national recommendation on sweet selling." Scandinavian Journal of Public Health **47**(5): 576-582. | 2019 |
| 10 | Arat, A., et al. (2020). "Organisation of preventive child health services: Key to socio-economic equity in vaccine uptake?" Scandinavian Journal of Public Health **48**(5): 491-494. | 2020 |
| 11 | Avlund, K., et al. (2003). "Social position and health in old age: the relevance of different indicators of social position." Scandinavian Journal of Public Health **31**(2): 126-136. | 2003 |
| 12 | Bagher, A., et al. (2016). "Socio-economic status and major trauma in a Scandinavian urban city: A population-based case-control study." Scandinavian Journal of Public Health **44**(2): 217-223. | 2016 |
| 13 | Balaj, M., et al. (2017). "Non-communicable diseases and the social determinants of health in the Nordic countries: Findings from the European Social Survey (2014) special module on the social determinants of health." Scandinavian Journal of Public Health **45**(2): 90-102. | 2017 |
| 14 | Bast, L. S., et al. (2021). "Socio-economic differences in smoking among adolescents in a school-based smoking intervention: The X:IT II study." Scandinavian Journal of Public Health **49**(8): 961-969. | 2021 |
| 15 | Blank, N. and B. Burstrom (2002). "Limiting long-term illness and the experience of financial strain in Sweden." Scandinavian Journal of Public Health **30**(1): 41-46. | 2002 |
| 16 | Bloomfield, K., et al. (2019). "Area-level relative deprivation and alcohol use in Denmark: Is there a relationship?" Scandinavian Journal of Public Health **47**(4): 428-438. | 2019 |
| 17 | Bohlin, A., et al. (2013). "Perceived gender inequality in the couple relationship and musculoskeletal pain in middle-aged women and men." Scandinavian Journal of Public Health **41**(8): 825-831. | 2013 |
| 18 | Brannlund, A. and A. Hammarstrom (2014). "Higher education and psychological distress: A 27-year prospective cohort study in Sweden." Scandinavian Journal of Public Health **42**(2): 155-162. | 2014 |
| 19 | Bremberg, S. (2020). "Rural-urban mortality inequalities in four Nordic welfare states." Scandinavian Journal of Public Health **48**(8): 791-793. | 2020 |
| 20 | Bringedal, B. and P. A. Tufte (2012). "Social and diagnostic inequality in health." Scandinavian Journal of Public Health **40**(7): 656-662. | 2012 |
| 21 | Bronnum-Hansen, H. (2000). "Socioeconomic differences in health expectancy in Denmark." Scandinavian Journal of Public Health **28**(3): 194-199. | 2000 |
| 22 | Bronnum-Hansen, H. and M. Baadsgaard (2008). "Increase in social inequality in health expectancy in Denmark." Scandinavian Journal of Public Health **36**(1): 44-51. | 2008 |
| 23 | Bronnum-Hansen, H., et al. (2017). "Persistent social inequality in life expectancy and disability-free life expectancy: Outlook for a differential pension age in Denmark?" Scandinavian Journal of Public Health **45**(4): 459-462. | 2017 |
| 24 | Bronnum-Hansen, H., et al. (2020). "Occupational inequality in health expectancy in Denmark." Scandinavian Journal of Public Health **48**(3): 338-345. | 2020 |
| 25 | Bruusgaard, D., et al. (2010). "Education and disability pension: A stronger association than previously found." Scandinavian Journal of Public Health **38**(7): 686-690. | 2010 |
| 26 | Cantarero-Arevalo, L., et al. (2016). "Ethnic differences in asthma treatment among Swedish adolescents: A multilevel analysis of individual heterogeneity." Scandinavian Journal of Public Health **44**(2): 184-194. | 2016 |
| 27 | Corell, M., et al. (2021). "Subjective health complaints in early adolescence reflect stress: A study among adolescents in Western Sweden." Scandinavian Journal of Public Health. | 2021 |
| 28 | Cubbin, C., et al. (2006). "Neighborhood deprivation and cardiovascular disease risk factors: Protective and harmful effects." Scandinavian Journal of Public Health **34**(3): 228-237. | 2006 |
| 29 | Dahl, E. and J. I. Elstad (2001). "Recent changes in social structure and health inequalities in Norway." Scandinavian Journal of Public Health: 7-17. | 2001 |
| 30 | Dalen, J. D., et al. (2012). "Are there educational differences in the association between self-rated health and mortality in Norway? The HUNT Study." Scandinavian Journal of Public Health **40**(7): 641-647. | 2012 |
| 31 | Doku, D. T., et al. (2020). "Social determinants of adolescent smoking over three generations." Scandinavian Journal of Public Health **48**(6): 646-656. | 2020 |
| 32 | Due, P., et al. (2019). "Increasing prevalence of emotional symptoms in higher socioeconomic strata: Trend study among Danish schoolchildren 1991-2014." Scandinavian Journal of Public Health **47**(7): 690-694. | 2019 |
| 33 | Dunlavy, A., et al. (2021). "Health outcomes in young adulthood among former child refugees in Denmark, Norway and Sweden: A cross-country comparative study." Scandinavian Journal of Public Health. | 2021 |
| 34 | Eek, F. and A. Axmon (2015). "Gender inequality at home is associated with poorer health for women." Scandinavian Journal of Public Health **43**(2): 176-182. | 2015 |
| 35 | Eek, F. and P. O. Ostergren (2009). "Factors associated with BMI change over five years in a Swedish adult population. Results from the Scania Public Health Cohort Study." Scandinavian Journal of Public Health **37**(5): 532-544. | 2009 |
| 36 | Egan, K. K., et al. (2021). "Association between alcohol, socioeconomic position and labour market participation: A prospective cohort study of transitions between work and unemployment." Scandinavian Journal of Public Health **49**(2): 197-205. | 2021 |
| 37 | Elstad, J. I. (2018). "Educational inequalities in hospital care for mortally ill patients in Norway." Scandinavian Journal of Public Health **46**(1): 74-82. | 2018 |
| 38 | Elwer, S., et al. (2015). "Life course models of economic stress and poor mental health in mid-adulthood: Results from the prospective Northern Swedish Cohort." Scandinavian Journal of Public Health **43**(8): 833-840. | 2015 |
| 39 | Erikson, R. and J. Torssander (2009). "Clerics die, doctors survive: A note on death risks among highly educated professionals." Scandinavian Journal of Public Health **37**(3): 227-231. | 2009 |
| 40 | Ernstsen, L., et al. (2010). "Educational inequalities in ischaemic heart disease mortality in 44,000 Norwegian women and men: The influence of psychosocial and behavioural factors. The HUNT Study." Scandinavian Journal of Public Health **38**(7): 678-685. | 2010 |
| 41 | Farrants, K. and C. Bambra (2018). "Neoliberalism and the recommodification of health inequalities: A case study of the Swedish welfare state 1980 to 2011." Scandinavian Journal of Public Health **46**(1): 18-26. | 2018 |
| 42 | Feveile, H., et al. (2011). "Industrial differences in female fertility treatment rates - A new approach to assess differences related to occupation?" Scandinavian Journal of Public Health **39**(2): 164-171. | 2011 |
| 43 | Fors, S., et al. (2011). "Live long and prosper? Childhood living conditions, marital status, social class in adulthood and mortality during mid-life: A cohort study." Scandinavian Journal of Public Health **39**(2): 179-186. | 2011 |
| 44 | Fors, S., et al. (2013). "Paying the price? The impact of smoking and obesity on health inequalities in later life." Scandinavian Journal of Public Health **41**(2): 134-141. | 2013 |
| 45 | Forssas, E., et al. (2010). "Socioeconomic differences in mortality among diabetic people in Finland." Scandinavian Journal of Public Health **38**(7): 691-698. | 2010 |
| 46 | Fritzell, J., et al. (2004). "The impact of income: assessing the relationship between income and health in Sweden." Scandinavian Journal of Public Health **32**(1): 6-16. | 2004 |
| 47 | Gisselmann, M. D. (2005). "Education, infant mortality, and low birth weight in Sweden 1973-1990: Emergence of the low birth weight paradox." Scandinavian Journal of Public Health **33**(1): 65-71. | 2005 |
| 48 | Gjesdal, S., et al. (2009). "Mortality among disability pensioners in Norway and Sweden 1990-96: Comparative prospective cohort study." Scandinavian Journal of Public Health **37**(2): 168-175. | 2009 |
| 49 | Graff-Iversen, S., et al. (2019). "Educational inequalities in midlife risk factors for non-communicable diseases in two Norwegian counties 1974-2002." Scandinavian Journal of Public Health **47**(7): 705-712. | 2019 |
| 50 | Granstrom, F., et al. (2015). "Exploring trends in and determinants of educational inequalities in self-rated health." Scandinavian Journal of Public Health **43**(7): 677-686. | 2015 |
| 51 | Groth, M. V., et al. (2014). "Disparities in dietary habits and physical activity in Denmark and trends from 1995 to 2008." Scandinavian Journal of Public Health **42**(7): 611-620. | 2014 |
| 52 | Gudmundsdottir, G. and R. Vilhjalmsson (2010). "Group differences in outpatient help-seeking for psychological distress: Results from a national prospective study of Icelanders." Scandinavian Journal of Public Health **38**(2): 160-167. | 2010 |
| 53 | Gundgaard, J. (2006). "Income-related inequality in utilization of health services in Denmark: Evidence from Funen County." Scandinavian Journal of Public Health **34**(5): 462-471. | 2006 |
| 54 | Gunnarsdottir, H., et al. (2014). "Time pressure among parents in the Nordic countries: A population-based cross-sectional study." Scandinavian Journal of Public Health **42**(2): 137-145. | 2014 |
| 55 | Hagen, K., et al. (2005). "Low socioeconomic status is associated with chronic musculoskeletal complaints among 46,901 adults in Norway." Scandinavian Journal of Public Health **33**(4): 268-275. | 2005 |
| 56 | Hammarstrom, A. and S. P. Phillips (2012). "Gender inequity needs to be regarded as a social determinant of depressive symptoms: Results from the Northern Swedish cohort." Scandinavian Journal of Public Health **40**(8): 746-752. | 2012 |
| 57 | Hammarstrom, A., et al. (2011). "It's no surprise! Men are not hit more than women by the health consequences of unemployment in the Northern Swedish Cohort." Scandinavian Journal of Public Health **39**(2): 187-193. | 2011 |
| 58 | Hansson, L. M., et al. (2010). "Perceived discrimination among men and women with normal weight and obesity. A population-based study from Sweden." Scandinavian Journal of Public Health **38**(6): 587-596. | 2010 |
| 59 | Haraldsdottir, S., et al. (2014). "Poorer self-rated health in residential areas with limited healthcare supply." Scandinavian Journal of Public Health **42**(3): 310-318. | 2014 |
| 60 | Haraldsdottir, S., et al. (2017). "Regional differences in mortality, hospital discharges and primary care contacts for cardiovascular disease." Scandinavian Journal of Public Health **45**(3): 260-268. | 2017 |
| 61 | Harkonmaki, K. et al. (2006) "Mental health functioning (SF-36) and intentions to retire early among ageing municipal employees: The Helsinki health study.” Scandinavian Journal of Public Health. **34**(2): 190-198. |  |
| 62 | Hemstrom, O. (2005). "Does high income buffer the association between adverse working conditions and ill health?" Scandinavian Journal of Public Health **33**(2): 131-137. | 2005 |
| 63 | Henriksson, G., et al. (2006). "Income distribution and mortality: Implications from a comparison of individual-level analysis and multilevel analysis with Swedish data." Scandinavian Journal of Public Health **34**(3): 287-294. | 2006 |
| 64 | Hetemaa, T., et al. (2006). "Socioeconomic inequities in invasive cardiac procedures among patients with incident angina pectoris or myocardial infarction." Scandinavian Journal of Public Health **34**(2): 116-123. | 2006 |
| 65 | Hewitt, S. and S. Graff-Iversen (2009). "Risk factors for cardiovascular diseases and diabetes in disability pensioners aged 40-42 years: A cross-sectional study in Norway." Scandinavian Journal of Public Health **37**(3): 280-286. | 2009 |
| 66 | Hjern, A. (2004). "Illicit drug abuse in second-generation immigrants: a register study in a national cohort of Swedish residents." Scandinavian Journal of Public Health **32**(1): 40-46. | 2004 |
| 67 | Holland, P., et al. (2009). "Socioeconomic inequalities in the employment impact of ischaemic heart disease: a longitudinal record linkage study in Sweden." Scandinavian Journal of Public Health **37**(5): 450-458. | 2009 |
| 68 | Holstein, B. E., et al. (2020). "Trends in socioeconomic differences in daily smoking among 15-year-old Danes 1991-2014." Scandinavian Journal of Public Health **48**(6): 667-673. | 2020 |
| 69 | Hyde, M., et al. (2004). "Bridges, pathways and valleys: labour market position and risk of hospitalization in a Swedish sample aged 55-63." Scandinavian Journal of Public Health **32**(5): 368-373. | 2004 |
| 70 | Janzon, E., et al. (2005). "Who are the "quitters''? a cross-sectional study of circumstances associated with women giving up smoking." Scandinavian Journal of Public Health **33**(3): 175-182. | 2005 |
| 71 | Johansen, A., et al. (2006). "Health behaviour among adolescents in Denmark: Influence of school class and individual risk factors." Scandinavian Journal of Public Health **34**(1): 32-40. | 2006 |
| 72 | Junna, L. M., et al. (2021). "Exploring the longevity advantage of doctorates in Finland and Sweden: The role of smoking- and alcohol-related causes of death." Scandinavian Journal of Public Health **49**(4): 419-422. | 2021 |
| 73 | Kaks, P. and M. Malqvist (2021). "Using an urban child health index to detect intra-urban disparities in Sweden." Scandinavian Journal of Public Health **49**(5): 563-570. | 2021 |
| 74 | Kark, M. and F. Rasmussen (2005). "Growing social inequalities in the occurrence of overweight and obesity among young men in Sweden." Scandinavian Journal of Public Health **33**(6): 472-477. | 2005 |
| 75 | Kelfve, S., et al. (2021). "Educational differences in long-term care use in Sweden during the last two years of life." Scandinavian Journal of Public Health. | 2021 |
| 76 | Kjollesdal, M. K. R., et al. (2016). "Educational differences in cardiovascular mortality: The role of shared family factors and cardiovascular risk factors." Scandinavian Journal of Public Health **44**(8): 744-750. | 2016 |
| 77 | Kluwer, B., et al. (2021). "Influenza risk groups in Norway by education and employment status." Scandinavian Journal of Public Health. | 2021 |
| 78 | Kristensen, P., et al. (2021). "Work participation in young Norwegians: a 19-year follow up in a registry-based life-course cohort." Scandinavian Journal of Public Health **49**(2): 176-187. | 2021 |
| 79 | Kristensen, T. S., et al. (2002). "Socioeconomic status and psychosocial work environment: results from a Danish national study." Scandinavian Journal of Public Health **30**(3): 41-48. | 2002 |
| 80 | Kristenson, M., et al. (2011). "Socioeconomic differences in outpatient healthcare utilisation are mainly seen for musculoskeletal problems in groups with poor self-rated health." Scandinavian Journal of Public Health **39**(8): 805-812. | 2011 |
| 81 | Krokstad, S. and S. Westin (2002). "Health inequalities by socioeconomic status among men in the Nord-Trondelag Health Study, Norway." Scandinavian Journal of Public Health **30**(2): 113-124. | 2002 |
| 82 | Laftman, S. B., et al. (2014). "Joint physical custody, turning to parents for emotional support, and subjective health: A study of adolescents in Stockholm, Sweden." Scandinavian Journal of Public Health **42**(5): 456-462. | 2014 |
| 83 | Lallukka, T., et al. (2015). "Economic difficulties and subsequent disability retirement." Scandinavian Journal of Public Health **43**(2): 169-175. | 2015 |
| 84 | Larsen, F. B., et al. (2021). "Population differences in health-related quality of life between cancer survivors and controls: Does low educational attainment widen the gap?" Scandinavian Journal of Public Health **49**(8): 821-832. | 2021 |
| 85 | Lehto, E., et al. (2013). "The role of psychosocial factors in socioeconomic differences in physical activity: A population-based study." Scandinavian Journal of Public Health **41**(6): 553-559. | 2013 |
| 86 | Leinonen, T., et al. (2012). "Interrelationships between education, occupational social class, and income as determinants of disability retirement." Scandinavian Journal of Public Health **40**(2): 157-166. | 2012 |
| 87 | Lindeus, M., et al. (2020). "Educational inequalities in fracture-related mortality using multiple cause of death data in the Skane region, Sweden." Scandinavian Journal of Public Health **48**(1): 72-79. | 2020 |
| 88 | Lindholm, C., et al. (2001). "Does chronic illness cause adverse social and economic consequences among Swedes?" Scandinavian Journal of Public Health **29**(1): 63-70. | 2001 |
| 89 | Lindstrom, C., et al. (2017). "Socioeconomic status, social capital and self-reported unmet health care needs: A population-based study." Scandinavian Journal of Public Health **45**(3): 212-221. | 2017 |
| 90 | Lindstrom, C., et al. (2020). "Unmet health-care needs and mortality: A prospective cohort study from southern Sweden." Scandinavian Journal of Public Health **48**(3): 267-274. | 2020 |
| 91 | Lissau, I., et al. (2001). "Social differences in illness and health-related exclusion from the labour market in Denmark from 1987 to 1994." Scandinavian Journal of Public Health: 19-30. | 2001 |
| 92 | Liu, Y., et al. (2018). "Socioeconomic differences in the use of alcohol and drunkenness in adolescents: Trends in the Health Behaviour in School-aged Children study in Finland 1990-2014." Scandinavian Journal of Public Health **46**(1): 102-111. | 2018 |
| 93 | Loyland, B., et al. (2021). "Differences in income trajectories according to psychological distress and pain: A longitudinal study among Norwegian social assistance recipients." Scandinavian Journal of Public Health **49**(8): 865-874. | 2021 |
| 94 | Lundberg, O., et al. (2001). "Changing health inequalities in a changing society? Sweden in the mid-1980s and mid-1990s." Scandinavian Journal of Public Health: 31-39. | 2001 |
| 95 | Madsen, A. A. (2020). "Return to work after first incidence of long-term sickness absence: A 10-year prospective follow-up study identifying labour-market trajectories using sequence analysis." Scandinavian Journal of Public Health **48**(2): 134-143. | 2020 |
| 96 | Maki, N. E. and P. T. Martikainen (2007). "Socioeconomic differences in suicide mortality by sex in Finland in 1971-2000: A register-based study of trends, levels, and life expectancy differences." Scandinavian Journal of Public Health **35**(4): 387-395. | 2007 |
| 97 | Manderbacka, K., et al. (2001). "Structural changes and social inequalities in health in Finland, 1986-1994." Scandinavian Journal of Public Health: 41-54. | 2001 |
| 98 | Manderbacka, K., et al. (2009). "Change and persistence in healthcare inequities: Access to elective surgery in Finland in 1992-2003." Scandinavian Journal of Public Health **37**(2): 131-138. | 2009 |
| 99 | Manderbacka, K., et al. (2015). "National and regional trends in equity within specialised health care in Finland in 2002-2010." Scandinavian Journal of Public Health **43**(5): 514-517. | 2015 |
| 100 | Martensson, S., et al. (2012). "Does participation in preventive child health care at the general practitioner minimise social differences in the use of specialist care outside the hospital system?" Scandinavian Journal of Public Health **40**(4): 316-324. | 2012 |
| 101 | Martikainen, P. et al. (2007) “Does survey non-response bias the association between occupational social class and health?” Scandinavian Journal of Public Health 35(2): 212-215. | 2007 |
| 102 | Matthiessen, J., et al. (2014). "Trends in overweight and obesity in Danish children and adolescents: 2000-2008-exploring changes according to parental education." Scandinavian Journal of Public Health **42**(4): 385-392. | 2014 |
| 103 | Meijer, M., et al. (2013). "A socioeconomic deprivation index for small areas in Denmark." Scandinavian Journal of Public Health **41**(6): 560-569. | 2013 |
| 104 | Molarius, A. and M. Hasselgren (2021). "Socioeconomic status, lifestyle factors and asthma prevalence: results from a population-based study in Sweden." Scandinavian Journal of Public Health. | 2021 |
| 105 | Moller, S. P., et al. (2020). "Socio-economic disparity in risk of undergoing emergency laparotomy and postoperative mortality." Scandinavian Journal of Public Health **48**(3): 250-258. | 2020 |
| 106 | Mols, R. E., et al. (2013). "Social factors and coping status in asymptomatic middle-aged Danes: Association to coronary artery calcification." Scandinavian Journal of Public Health **41**(7): 737-743. | 2013 |
| 107 | Mortensen, L. H., et al. (2010). "Income-related and educational inequality in small-for-gestational age and preterm birth in Denmark and Finland 1987-2003." Scandinavian Journal of Public Health **38**(1): 40-45. | 2010 |
| 108 | Moussa, K. M., et al. (2004). "Socioeconomic and demographic differences in exposure to environmental tobacco smoke at work: the Scania Public Health Survey 2000." Scandinavian Journal of Public Health **32**(3): 194-202. | 2004 |
| 109 | Nielsen, L., et al. (2015). "Socioeconomic differences in emotional symptoms among adolescents in the Nordic countries: Recommendations on how to present inequality." Scandinavian Journal of Public Health **43**(1): 83-90. | 2015 |
| 110 | Nielsen, S. S., et al. (2012). "Is there equity in use of healthcare services among immigrants, their descendents, and ethnic Danes?" Scandinavian Journal of Public Health **40**(3): 260-270. | 2012 |
| 111 | Nilsen, S. A., et al. (2020). "Complex families and health complaints among adolescents: A population-based cross-sectional study." Scandinavian Journal of Public Health **48**(7): 733-742. | 2020 |
| 112 | Nilsen, S. M., et al. (2012). "Educational inequalities in disability pensioning - the impact of illness and occupational, psychosocial, and behavioural factors: The Nord-Trondelag Health Study (HUNT)." Scandinavian Journal of Public Health **40**(2): 133-141. | 2012 |
| 113 | Nilsson, P. M., et al. (2005). "Social mobility, marital status, and mortality risk in an adult life course perspective: The Malmo preventive project." Scandinavian Journal of Public Health **33**(6): 412-423. | 2005 |
| 114 | Norredam, M., et al. (2004). "Emergency room utilization in Copenhagen: a comparison of immigrant groups and Danish-born residents." Scandinavian Journal of Public Health **32**(1): 53-59. | 2004 |
| 115 | Nummela, O. P., et al. (2007). "Self-rated health and indicators of SES among the ageing in three types of communities." Scandinavian Journal of Public Health **35**(1): 39-47. | 2007 |
| 116 | Nyqvist, F., et al. (2013). "A comparison of older workers' and retired older people's social capital and sense of mastery." Scandinavian Journal of Public Health **41**(8): 792-798. | 2013 |
| 117 | Oftedal, A. M., et al. (2016). "Socio-economic risk factors for preterm birth in Norway 1999-2009." Scandinavian Journal of Public Health **44**(6): 587-592. | 2016 |
| 118 | Ostergren, O. (2015). "Growing gaps: The importance of income and family for educational inequalities in mortality among Swedish men and women 1990-2009." Scandinavian Journal of Public Health **43**(6): 563-570. | 2015 |
| 119 | Padyab, M., et al. (2013). "Life course socioeconomic position and mortality: A population register-based study from Sweden." Scandinavian Journal of Public Health **41**(8): 785-791. | 2013 |
| 120 | Pape, K., et al. (2013). "Medical benefits in young Norwegians and their parents, and the contribution of family health and socioeconomic status. The HUNT Study, Norway." Scandinavian Journal of Public Health **41**(5): 455-462. | 2013 |
| 121 | Pedersen, P. V., et al. (2009). "Readiness to change level of physical activity in leisure time among physically inactive Danish adults." Scandinavian Journal of Public Health **37**(8): 785-792. | 2009 |
| 122 | Pennanen, M., et al. (2011). "Academic achievement and smoking: Is self-efficacy an important factor in understanding social inequalities in Finnish adolescents?" Scandinavian Journal of Public Health **39**(7): 714-722. | 2011 |
| 123 | Pensola, T. H. and P. Martikainen (2003). "Effect of living conditions in the parental home and youth paths on the social class differences in mortality among women." Scandinavian Journal of Public Health **31**(6): 428-438. | 2003 |
| 124 | Perhoniemi, R., et al. (2020). "Determinants of disability pension applications and awarded disability pensions in Finland, 2009 and 2014." Scandinavian Journal of Public Health **48**(2): 172-180. | 2020 |
| 125 | Petersen, C. B., et al. (2010). "Time trends in physical activity in leisure time in the Danish population from 1987 to 2005." Scandinavian Journal of Public Health **38**(2): 121-128. | 2010 |
| 126 | Pietilainen, O., et al. (2018). "Occupational class inequalities in disability retirement after hospitalisation." Scandinavian Journal of Public Health **46**(3): 331-339. | 2018 |
| 127 | Piha, K., et al. (2007). "Trends in socioeconomic differences in sickness absence among Finnish municipal employees 1990-99." Scandinavian Journal of Public Health **35**(4): 348-355. | 2007 |
| 128 | Polvinen, A., et al. (2015). "Socioeconomic inequalities in cause-specific mortality after disability retirement due to different diseases." Scandinavian Journal of Public Health **43**(2): 159-168. | 2015 |
| 129 | Poulsen, K. and L. L. Andersen (2016). "Linking data on work, health and lifestyle to explain socio-occupational inequality in Danish register-based incidence of diabetes." Scandinavian Journal of Public Health **44**(4): 361-368. | 2016 |
| 130 | Poulsen, K., et al. (2014). "Diabetes and work: 12-year national follow-up study of the association of diabetes incidence with socioeconomic group, age, gender and country of origin." Scandinavian Journal of Public Health **42**(8): 728-733. | 2014 |
| 131 | Povlsen, L. (2012) “Immigrant women’s clubs in a health-¨promotion perspective.” Scandinavian Journal of Public Health 40(4): 355-359. | 2012 |
| 132 | Povlsen, L., et al. (2018). "Economic poverty among children and adolescents in the Nordic countries." Scandinavian Journal of Public Health **46**: 30-37. | 2018 |
| 133 | Ramadani, R. V., et al. (2019). "The moderating effect of income on the relationship between body mass index and health-related quality of life in Northern Sweden." Scandinavian Journal of Public Health **47**(7): 765-773. | 2019 |
| 134 | Rasmussen, M., et al. (2009). "Social inequality in adolescent daily smoking: Has it changed over time?" Scandinavian Journal of Public Health **37**(3): 287-294. | 2009 |
| 135 | Rautio, N., et al. (2011). "Socioeconomic position and effectiveness of lifestyle intervention in prevention of type 2 diabetes: One-year follow-up of the FIN-D2D project." Scandinavian Journal of Public Health **39**(6): 561-570. | 2011 |
| 136 | Rayce, S. L., et al. (2008). "Economic consequences of incident disease: The effect on loss of annual income." Scandinavian Journal of Public Health **36**(3): 258-264. | 2008 |
| 137 | Roos, E., et al. (2005). "The association of employment status and family status with health among women and men in four Nordic countries." Scandinavian Journal of Public Health **33**(4): 250-260. | 2005 |
| 138 | Rostad, B., et al. (2009). "Social inequalities in mortality in older women cannot be explained by biological and health behavioural factors - results from a Norwegian health survey (the HUNT Study)." Scandinavian Journal of Public Health **37**(4): 401-408. | 2009 |
| 139 | Ruokolainen, O., et al. (2021). "Association between educational level and smoking cessation in an 11-year follow-up study of a national health survey." Scandinavian Journal of Public Health **49**(8): 951-960. | 2021 |
| 140 | Saeter, S.M.M., et al. (2018) " Health complaints in late adolescence; Frequency, factor structure and the association with socio-economic status." Scandinavian Journal of Public Health. 46(1): 141-149. | 2018 |
| 141 | Samdal, G. B., et al. (2019). "The Norwegian Healthy Life Centre Study: A pragmatic RCT of physical activity in primary care." Scandinavian Journal of Public Health **47**(1): 18-27. | 2019 |
| 142 | San Sebastian, M., et al. (2020). "Rural-urban differences in suicide attempts and mortality among young people in northern Sweden, 1998-2017: A register-based study." Scandinavian Journal of Public Health **48**(8): 794-800. | 2020 |
| 143 | Sigurdardottir, A. K., et al. (2013). "Socioeconomic status and differences in medication use among older people according to ATC categories and urban-rural residency." Scandinavian Journal of Public Health **41**(3): 311-317. | 2013 |
| 144 | Siven, S. S. E., et al. (2015). "Social, lifestyle and demographic inequalities in hypertension care." Scandinavian Journal of Public Health **43**(3): 246-253. | 2015 |
| 145 | Sondergaard, G., et al. (2018). "Educational inequality in cardiovascular diseases: a sibling approach." Scandinavian Journal of Public Health **46**(1): 83-91. | 2018 |
| 146 | Sortso, C., et al. (2018). "Social inequality in diabetes patients' morbidity patterns from diagnosis to death - A Danish register-based investigation." Scandinavian Journal of Public Health **46**(1): 92-101. | 2018 |
| 147 | Spein, A. R., et al. (2004). "Predictors of smoking behaviour among indigenous Sami adolescents and non-indigenous peers in North Norway." Scandinavian Journal of Public Health **32**(2): 118-129. | 2004 |
| 148 | Stenmark, H., et al. (2016). "Mental problems and their socio-demographic determinants in young schoolchildren in Sweden, a country with high gender and income equality." Scandinavian Journal of Public Health **44**(1): 18-26. | 2016 |
| 149 | Stickley, A. and P. Carlson (2010). "Factors associated with non-lethal violent victimization in Sweden in 2004-2007." Scandinavian Journal of Public Health **38**(4): 404-410. | 2010 |
| 150 | Storeng, S. H., et al. (2018). "Decennial trends and inequalities in healthy life expectancy: The HUNT Study, Norway." Scandinavian Journal of Public Health **46**(1): 124-131. | 2018 |
| 151 | Storeng, S. H., et al. (2021). "Trends in Disability-Free Life Expectancy (DFLE) from 1995 to 2017 in the older Norwegian population by sex and education: The HUNT Study." Scandinavian Journal of Public Health. | 2021 |
| 152 | Sundberg, L., et al. (2021). "Increasing inequalities in disability-free life expectancy among older adults in Sweden 2002-2014." Scandinavian Journal of Public Health. | 2021 |
| 153 | Szilcz, M., et al. (2018). "Time trends in absolute and relative socioeconomic inequalities in leisure time physical inactivity in northern Sweden." Scandinavian Journal of Public Health **46**(1): 112-123. | 2018 |
| 154 | Szilcz, M., et al. (2020). "Income inequalities in leisure time physical inactivity in northern Sweden: A decomposition analysis." Scandinavian Journal of Public Health **48**(4): 442-451. | 2020 |
| 155 | Tiikkaja, S. and O. Hemstrem (2008). "Does intergenerational social mobility among men affect cardiovascular mortality? A population-based register study from Sweden." Scandinavian Journal of Public Health **36**(6): 619-628. | 2008 |
| 156 | Toivanen, S. (2011). "Income differences in stroke mortality: A 12-year follow-up study of the Swedish working population." Scandinavian Journal of Public Health **39**(8): 797-804. | 2011 |
| 157 | Torsheim, T., et al. (2018). "Social inequalities in self-rated health: A comparative cross-national study among 32,560 Nordic adolescents." Scandinavian Journal of Public Health **46**(1): 150-156. | 2018 |
| 158 | Upmark, M., et al. (2001). "Conditions during childhood and adolescence as explanations of social class differences in disability pension among young men." Scandinavian Journal of Public Health **29**(2): 96-103. | 2001 |
| 159 | Vikum, E., et al. (2012). "Socioeconomic inequalities in dental services utilisation in a Norwegian county: The third Nord-Trondelag Health Survey." Scandinavian Journal of Public Health **40**(7): 648-655. | 2012 |
| 160 | Vilhjalmsson, R. (2021). "Family income and insufficient medical care: A prospective study of alternative explanations." Scandinavian Journal of Public Health **49**(8): 875-883. | 2021 |
| 161 | Volanen, S. M., et al. (2006). "Sense of coherence and its determinants: A comparative study of the Finnish-speaking majority and the Swedish-speaking minority in Finland." Scandinavian Journal of Public Health **34**(5): 515-525. | 2006 |
| 162 | Voutilainen, A., et al. (2015). "Associations across spatial patterns of disease incidences, socio-demographics, and land use in Finland 1991-2010." Scandinavian Journal of Public Health **43**(4): 356-363. | 2015 |
| 163 | Waenerlund, A. K., et al. (2011). "Is temporary employment related to health status? Analysis of the Northern Swedish Cohort." Scandinavian Journal of Public Health **39**(5): 533-539. | 2011 |
| 164 | Waenerlund, A. K., et al. (2019). "Trends in educational and income inequalities in cardiovascular morbidity in middle age in Northern Sweden 1993-2010." Scandinavian Journal of Public Health **47**(7): 713-721. | 2019 |
| 165 | Wagenius, C. M., et al. (2019). "Access for all? Assessing vertical and horizontal inequities in healthcare utilization among young people in northern Sweden." Scandinavian Journal of Public Health **47**(1): 1-8. | 2019 |
| 166 | Wastesson, J. W., et al. (2014). "Inequalities in health care use among older adults in Sweden 1992-2011: A repeated cross-sectional study of Swedes aged 77 years and older." Scandinavian Journal of Public Health **42**(8): 795-803. | 2014 |
| 167 | Wemrell, M., et al. (2021). "Socio-economic disparities in the dispensation of antibiotics in Sweden 2016-2017: An intersectional analysis of individual heterogeneity and discriminatory accuracy." Scandinavian Journal of Public Health. | 2021 |
| 168 | Westin, M. and R. Westerling (2006). "Health and healthcare utilization among single mothers and single fathers in Sweden." Scandinavian Journal of Public Health **34**(2): 182-189. | 2006 |
| 169 | Westin, M. and R. Westerling (2007). "Social capital and inequality in health between single and couple parents in Sweden." Scandinavian Journal of Public Health **35**(6): 609-617. | 2007 |
| 170 | Winding, T. N., et al. (2013). "Personal predictors of educational attainment after compulsory school: Influence of measures of vulnerability, health, and school performance." Scandinavian Journal of Public Health **41**(1): 92-101. | 2013 |
| 171 | Yngwe, M. A., et al. (2006). "On the importance of internalized consumption norms for ill health." Scandinavian Journal of Public Health **34**(1): 76-82. | 2006 |
